# Fluorescence Biomodulation for Canine Interdigital Furunculosis: Updates for Once-Weekly Schedule

**DOI:** 10.3389/fvets.2022.880349

**Published:** 2022-06-20

**Authors:** Andrea Marchegiani, Alessandro Fruganti, Alessandra Gavazza, Andrea Spaterna, Matteo Cerquetella

**Affiliations:** School of Biosciences and Veterinary Medicine, University of Camerino, Camerino, Italy

**Keywords:** dog, interdigital furunculosis, fluorescence biomodulation, pododermatitis, photobiomodulation

## Abstract

Interdigital furunculosis is a common multifactorial, inflammatory disease of the canine interdigital skin in which lesions commonly become secondarily infected. Fluorescence biomodulation (FBM) administered twice weekly has shown to effectively control clinical manifestation as adjunct therapy to systemic antibiotic. Since twice weekly regimen could be unaffordable for some pet owners, the aim of this study was to evaluate the effect of once weekly application of FBM in combination with systemic antibiotic on clinical manifestations of canine interdigital pyoderma, comparing the results to those present in literature. Twelve dogs diagnosed with interdigital pyoderma received antibiotic plus once weekly FBM application. Dogs were scored until complete healing based on global lesion score and neutrophil engulfing bacterial score. The results obtained demonstrated that once weekly application of FBM exerts the same beneficial effect on interdigital furunculosis healing as per twice weekly, indicating that once weekly regimen is well tolerated and is yielding similar results to twice weekly applications.

## Introduction

Canine interdigital furunculosis (CIF) is an inflammatory and debilitating skin disease affecting one or more feet of dogs, representing a frequent diagnosis in canine dermatology (1). Despite conformational problem of the foot can predispose to deep bacterial infection of the pedal skin, CIF is usually triggered by an underlying disease such as atopy, cutaneous adverse food reaction, ectoparasites, endocrine disease, foreign bodies, or conformational problem of the foot (2, 3). Historically, the condition has been generally treated with prolonged courses of antibiotics and topical or systemic anti-inflammatory drugs to manage both the infection and the inflammation (4). Among non-pharmacological procedures explored in canine dermatology, promising results have been obtained using different forms of photobiomodulation, in which photons (mainly produced by light-emitting Diodes, LED) are administered at different wavelengths to influence biological activity and enhance cutaneous restoration from diseases (5). Photobiomodulation has been successfully applied in a variety of dermatological disorders such as pyotraumatic dermatitis, otitis externa, licking granulomas (6), pruritus (7), non-inflammatory alopecia (8), and pododermatitis (9). Fluorescent biomodulation (FBM) is an innovative form of PBM that uses fluorescence to induce different beneficial effect on affected tissues. FBM has been demonstrated to be able to statistically downregulate the expression of tumor necrosis factor alpha and upregulate epidermal growth factor, fibroblast growth factors, transforming growth factor beta, collagen I and III, Ki67, factor VIII, and decorin (DCN), in addition to determine an increase in both number and size of mitochondria (10, 11). Such novel technique has been successfully applied to manage various canine dermatological conditions as superficial bacterial folliculitis (5), deep pyoderma (12), canine perianal fistulas (13), and cutaneous calcinosis (14). In addition to these, FBM has been explored as adjunct care to systemic antibiotic in a clinical trial regarding CIF (15). Thirty-six dogs received either systemic oral antibiotics alone or antibiotics plus FBM application twice weekly until complete clinical healing from CIF, which occurred in a mean time of 10.4 ± 4.9 weeks (median, 10.0 weeks) and 4.3 ± 2.2 weeks (median, 3.5 weeks), respectively. In the real life, twice weekly veterinary visits could be unappealing or difficult for some pet owners and it would be desirable to reduce the visit frequency to gain and maintain owners' compliance. The aim of the present study is to investigate the effect of two consecutive FBM applications, done on a weekly visit plan, as an adjunction to systemic antibiotic on patients with interdigital furunculosis.

## Materials and Methods

The animal study was reviewed and approved by University of Camerino Ethical Committee for Animal Use. Written informed consent was obtained from the owners for the participation of their animals in this study. Twelve (12) dogs with diagnosis of CIF were enrolled in this study. In order to compare results obtained from the present study to those already available in literature, the same inclusion/exclusion criteria, blinding scheme, scoring system and clinical assessment were employed (15). Briefly, to be enrolled dogs had to present interdigital furunculosis scored according to a scoring system (global lesion score, GLS) for canine interdigital furunculosis (15). This system is based on the grading of characteristic lesion of CIF as (i) hemorrhagic vesicles and bullae; (ii) hemorrhagic crust and papules; (iii) fistula with draining tracts; (iv) ulcers and erosions; each scored on a 0–4 scale resulting in a global lesion score (GLS) range of 0–16. To be enrolled, dogs had to score 3 or 4 in one of these lesion types at least. For dogs that presented with a range of different lesions in the same paw, the entire foot was scored. Only one paw per dog was scored and always the same paw. When more than one paw was affected at enrolment, the most severely affected paw was selected for the study (the one scoring highest). To avoid any possible bias regarding the scoring of the lesions, the principal investigator (PI) examined the dogs on the day of enrolment and remained blinded on the treatment received until the end of the study. Th PI evaluated the dogs on a weekly basis using photographs uploaded online (Google Drive) by the collaborating investigator, who was not blinded and performed all fluorescence biomodulation applications. At enrolment, all dogs underwent culture and sensitivity sampling from pedal lesions; dogs whose isolates were tested to be resistant to all antibiotics were not enrolled in the study. As per previous evaluation (15), in the 2 weeks before and throughout the course of the study, systemic antihistamines, antibiotics, glucocorticoids, cyclosporine, oclacitinib, or additional topical treatments as anti-inflammatory or antimicrobials were not permitted. Feeding and housing conditions were left unchanged during the length of the study. *Demodex* and *Malassezia* infestation were ruled out before enrolment; blood and urine samples were obtained for rule out any systemic illness. Parasitic dermatoses, fungal/yeast infections, leishmaniasis, endocrine or metabolic disorders, allergic pruritus, cutaneous neoplasia, or kidney malfunction represented exclusion criteria. The use of concurrent flea control and shampoo without antimicrobial activity was permitted and recorded. In addition to systemic antibiotics therapy (cephalexin 20 mg/kg p.o q 12 h) all dogs received FBM procedure (Phovia™, Vetoquinol, France), which consists of applying an ~2 mm layer of the gel to the lesions and illuminating them with the blue LED device. This delivers non-coherent blue light with peak wavelength between 440 and 460 nm and power density between 55 and 129 mW/cm^2^, for 2 min, at ~5 cm distance. This protocol was applied once a week with two consecutive applications in the same session for each dog, with a one-minute rest between one illumination and the other. Dogs were considered healed when lesions were scored as 1 or 0, according to (15). Cytological assessment of stained impression smears was taken where an open lesion was present and neutrophils engulfing score (NES, 0–4) was calculated. The number of neutrophil engulfing bacteria per high powered field (x500 magnification) were counted and an average was taken over 10 microscopic fields, according to the following scheme: NES score equal to 0, 1, 2, 3, 4, if number of neutrophils engulfing bacteria was equal to none, <1, 1–4, 5–10, and >10, respectively (15). All data analyses (Fisher exact test and Wilcoxon–Mann–Whitney *U*-tests) were conducted using GraphPad Prism 8 and values of *P* ≤ 0.05 were considered significant.

## Results

**Figure 1:**
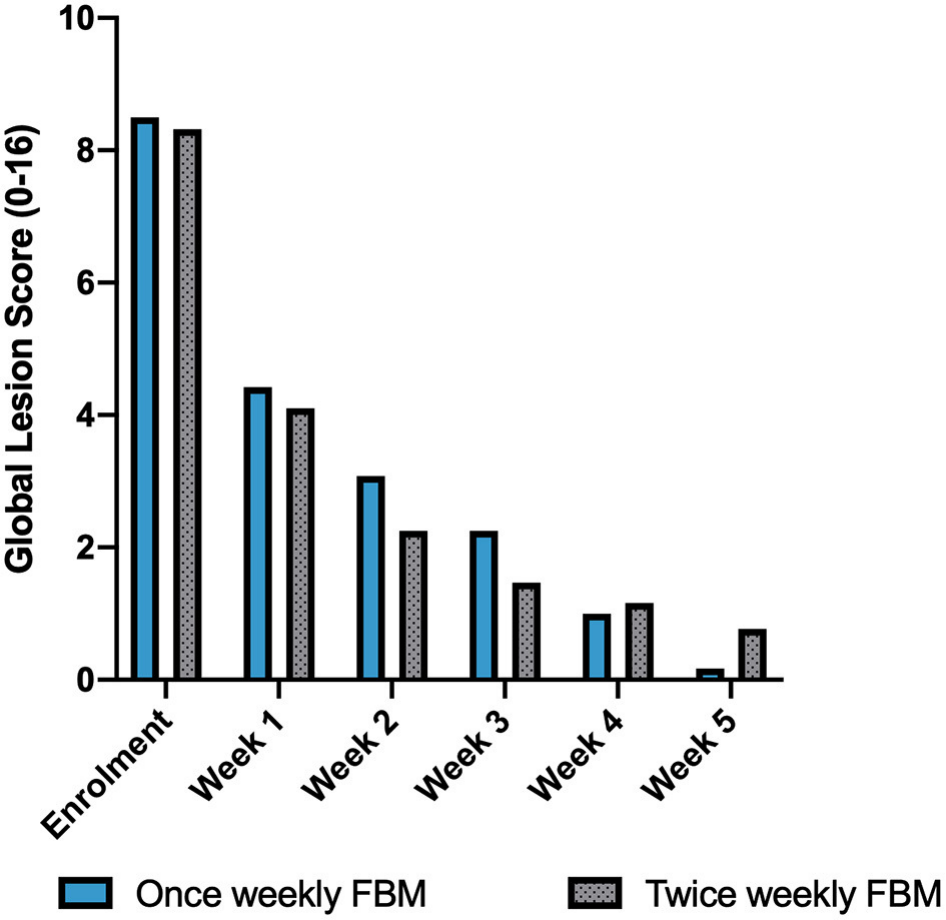
Global lesion score per week of study, in comparison with those reported for twice weekly FBM application from previous published study (15).

**Figure 2:**
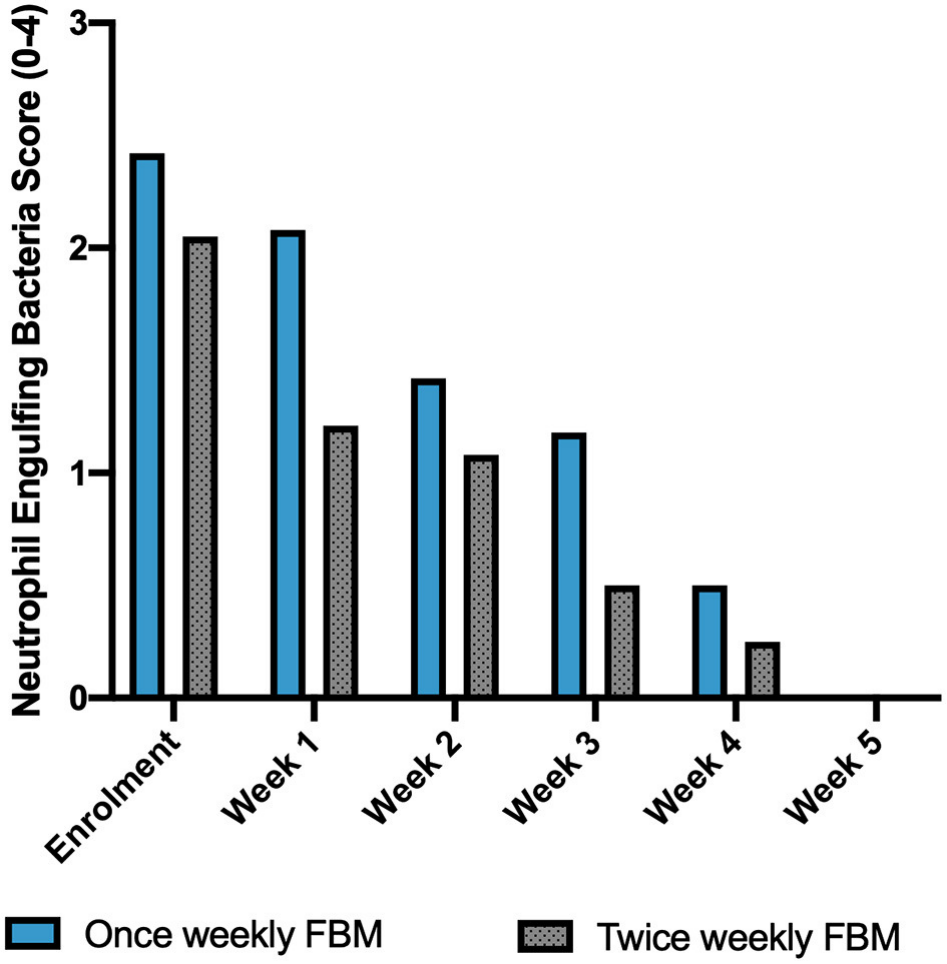
Average Neutrophil Engulfing Bacteria (NES) scores per week of study, in comparison with those reported for twice weekly FBM application from previous published study (15).

All twelve dogs that met inclusion and exclusion criteria were enrolled and all achieved clinical resolution and thus completed the study. Although this was not a controlled study, a comparison with the results obtained from twice weekly FBM application previously published (15) was done. No significant differences in sex distribution, age, and number of purebred dogs, were identified between once and twice weekly FBM study, as shown in Table 1. Microbiological swabs performed on lesions showed *Staphylococcus* spp. (*n* = 12) as predominant pathogen; other species found were *S. pseudintermedius* (9) and *S. aureus* (3). Several other different bacteria (*Streptococcus* spp., *Enterococcus* spp., and others) were also detected but less frequently. All the identified bacteria were susceptible to cefalexin and there was no need to change antibiotic. The average time to achieve complete clinical resolution with once-weekly regimen was 4.5 weeks, with a standard deviation of 0.5 weeks and a median of 4.5 weeks. Fisher exact test and Wilcoxon–Mann–Whitney *U*-tests showed no statistical difference between the once- or twice-weekly FBM (*p* = 0.81 for both tests). The GLS and NES variations over time are represented in Tables 2, 3, respectively. Table 4 compares the time to achieve clinical resolution with those obtained with the twice weekly FBM application (15). Tables 5, 6 (then Figures 1, 2) show a comparison between GLS and NES results from twice and once weekly FBM application; no statistical differences were seen between groups at any timepoint.

**Table 1:** Signalment data of dogs.

	**Current study**	**Twice weekly**
	**(once weekly)**	**study (15)**
Breed		
Mixed breed	7	11
Doberman	1	–
Labrador	3	2
German Shepherd	–	2
Pitbull	–	2
Bulldog	1	1
Italian Bracco	–	1
**Sex**
Male	10	15
Female	2	4
**Age (years)**
Mean ± sd	5.81 ± 2.87	5.42 ± 3.06
**Weight (kg)**
Mean	31.25	33.79
Sd	9.04	7.04
**Body condition score (BCS)**
Mean	5.3	5.7
Sd	0.4	0.7

*Those related to twice weekly FBM application are derived from previous published study (15)*.

**Table 2:** GLS values for once-weekly FBM application.

**Timepoints**	**Patient ID**												**Mean**	**Sd**
	**1**	**2**	**3**	**4**	**5**	**6**	**7**	**8**	**9**	**10**	**11**	**12**	
*E*	7	12	8	9	6	9	7	8	9	10	10	7	8.50	1.68
*w1*	6	5	5	6	4	3	5	2	3	5	6	3	4.42	1.38
*w2*	5	4	3	4	3	3	3	0	3	3	4	2	3.08	1.24
*w3*	3	3	2	3	2	2	2	2	2	2	2	2	2.25	0.45
*w4*	2	2	2	4	0	0	0	0	0	2	0	0	1.00	1.35
*w5*	1	0	0	1	0	0	0	0	0	0	0	0	0.17	0.39

**Table 3:** NES values for once-weekly FBM application.

**Timepoints**	**Patient ID**												**Mean**	**St. Dev**.
	**1**	**2**	**3**	**4**	**5**	**6**	**7**	**8**	**9**	**10**	**11**	**12**	
*E*	3	2	2	3	2	3	3	2	2	2	3	2	2.45	0.52
*w1*	3	3	2	2	2	2	2	1	2	2	2	2	2.09	0.54
*w2*	2	2	2	1	2	1	2	1	1	0	2	1	1.45	0.69
*w3*	2	3	0	2	3	1	1	0	0	-	1	0	1.30	1.16
*w4*	1	0	1	0	0	0	2	–	–	–	0	–	0.50	0.76
*w5*	0	–	0	–	–	–	0	–	–	–	–	–	0.00	0.00

**Table 4:** Summary of time to clinical resolution between once and twice weekly FBM application.

**FBM**	**Number of**	**Mean**	**Std. dev**.	**Median**
**regimen**	**patients**	**(weeks)**	**(weeks)**	**(weeks)**
Once weekly	12	4.5	0.5	4.5
(current study)
Twice weekly	19	4.3	2.2	3.5
(15)

*No statistical difference between FBM regimens was highlighted. Data related to twice weekly FBM application are derived from previous published study (15)*.

**Table 5:** Summary of GLS values between once and twice weekly FBM application.

**Global**	**Once**	**Twice**	***p*-**
**lesion**	**weekly**	**weekly**	**values**
**score**	**(current study)**	**(15)**	
Enrolment	8.50	8.32	0.28
Week 1/Day 7	4.42	4.10	0.54
Week 2/Day 14	3.08	2.25	0.95
Week 3/Day 21	2.25	1.47	0.29
Week 4/Day 28	1.00	1.16	0.98
Week 5/Day 35	0.17	0.77	0.68

*Data related to twice weekly FBM application are derived from previous published study (15)*.

**Table 6:** Summary of NES values between once and twice weekly FBM application.

**Neutrophils**	**Once**		**Twice**		***p*-**
**engulfing bacteria**	**weekly**		**weekly**		**values**
**score**	**(current study)**		**(** **15** **)**		
	***N***	**Mean**	***N***	**Mean**	
Enrolment	12	2.42	19	2.05	0.12
Week 1/Day 7	12	2.08	19	1.21	0.07
Week 2/Day 14	12	1.42	13	1.08	0.23
Week 3/Day 21	11	1.18	12	0.5	0.19
Week 4/Day 28	8	0.5	8	0.25	0.50
Week 5/Day 35	3	0	4	0	–

*Data related to twice weekly FBM application are derived from previous published study (15)*.

## Discussion and Conclusion

Interdigital furunculosis is a common and potentially debilitating diseases of dogs representing the second most frequent cause for presentation to first opinion veterinary practices in a United Kingdom (UK) survey on canine skin problems (16). This perpetuating nature of the condition is often frustrating for pet owners and can be worsened if there is poor compliance or adherence to treatment, because this is likely to compromise efficacy of the treatment protocol and influence recurrence of the condition (17). For the successful management of this condition it is essential to effectively manage the clinical manifestation and also to uncover and succeed the underlying primary disease or predisposing factors (18). Traditional therapy with systemic antibiotics often results in improved clinical symptoms after 2–4 weeks of treatment, but complete resolution often takes 4–6 weeks or longer. Prolonged antibiotic treatment (8–12 weeks) is often needed in cases of severe interdigital pyoderma, possibly decreasing the compliance of the owners (19). Dermatological conditions impacts on quality of life of dogs and their owners, which is profoundly affected also by the frequency of veterinary consultations, making twice weekly visits challenging for many pet owners (20–22). The results of the present study demonstrated that once weekly application of FBM exerts beneficial effect on interdigital furunculosis healing. FBM is currently applied in human dermatology for the treatment of acne (23) and two consecutive applications of fluorescent light energy administered once weekly is a protocol that has been used in a recent clinical study on wound healing, allowing to control both proinflammatory and anti-inflammatory factors as well as reduce the frequency of visits required and time needed to re-epithelization (24). Once weekly FBM application has been demonstrated to be effective in the management of canine perianal fistulas (13) and the results of the present study demonstrate that the once weekly FBM regimen (with two applications in the same session, with 1-min rest) provides a useful tool for CIF management, accelerating the time needed to achieve clinical resolution in comparison to traditional treatment modalities, in the same fashion of twice weekly single FBM application. These results indicate that the once weekly application regimen is well-tolerated and is yielding similar results to twice weekly applications. The findings of the present study confirm FBM aptitude in veterinary medicine that have been largely demonstrated in a variety of clinical condition involving both infectious and inflammatory diseases. In patients and owners who are not prepared to do twice weekly visits, the once weekly applications FBM schedule can be offered as a valid alternative in the management of CIF when used as an adjunct therapy to systemic antibiotics.

## Data Availability Statement

The raw data supporting the conclusions of this article will be made available by the authors, without undue reservation.

## Ethics Statement

The animal study was reviewed and approved by University of Camerino Ethical Committee for Animal Use. Written informed consent was obtained from the owners for the participation of their animals in this study.

## Author Contributions

AM and MC contributed to conception and design of the study. AM, AF, AG, and AS executed the procedures of the study. AM organized the database, performed the statistical analysis, and wrote the first draft of the manuscript. AS, AF, AG, and MC wrote sections of the manuscript. All authors contributed to manuscript revision, read, and approved the submitted version.

## Conflict of Interest

AM has received an honorarium from Vetoquinol Italy. The remaining authors declare that the research was conducted in the absence of any commercial or financial relationships that could be construed as a potential conflict of interest.

## Publisher's Note

All claims expressed in this article are solely those of the authors and do not necessarily represent those of their affiliated organizations, or those of the publisher, the editors and the reviewers. Any product that may be evaluated in this article, or claim that may be made by its manufacturer, is not guaranteed or endorsed by the publisher.

## References

[1] van den Broek A Horvath-Ungerboeck C. Pedal dermatitis Part 2: canine pododermatitis. Companion Anim. (2011) 16:41–6. 10.1111/j.2044-3862.2010.00022.x

[2] Breathnach RM Fanning S Mulcahy G Bassett HF Jones BR. Canine pododermatitis and idiopathic disease. Vet J. (2008) 176:146–57. 10.1016/j.tvjl.2007.05.027 17919951

[3] Duclos DD Hargis AM Hanley PW Breathnach RM Baker KP Quinn PJ. Canine pododermatitis. Vet Clin N Am Small Anim Pract. (2013) 43:57–87. 10.1016/j.cvsm.2012.09.012 23182325

[4] Bajwa J. Canine pododermatitis. Can Vet J. (2016) 57:991–3. 27587895 PMC4982575

[5] Marchegiani A Spaterna A Cerquetella M. Current applications and future perspectives of fluorescence light energy biomodulation in veterinary medicine. Ve Sci MDPI. (2021) 2021:20. 10.3390/vetsci8020020 33504091 PMC7912178

[6] Godine RL. Low level laser therapy (LLLT) in veterinary medicine. Photomed Laser Surg. (2014) 32:1–2. 10.1089/pho.2013.9867 24359266

[7] Stich AN Rosenkrantz WS Griffin CE. Clinical efficacy of low-level laser therapy on localized canine atopic dermatitis severity score and localized pruritic visual analog score in pedal pruritus due to canine atopic dermatitis. Vet Dermatol. (2014) 25:464–e74. 10.1111/vde.12144 24909192

[8] Olivieri L Cavina D Radicchi G Miragliotta V Abramo F. Efficacy of low-level laser therapy on hair regrowth in dogs with noninflammatory alopecia: a pilot study. Vet Dermatol. (2015) 26:35–e11. 10.1111/vde.12170 25227429

[9] Perego R Proverbio D Zuccaro A Spada E. Low-level laser therapy: case-control study in dogs with sterile pyogranulomatous pododermatitis. Vet World. (2016) 9:882–7. 10.14202/vetworld.2016.882-887 27651678 PMC5021839

[10] Hamblin MR. Photobiomodulation or low-level laser therapy. J Biophotonics. (2016) 9:1122–4. 10.1002/jbio.201670113 27973730 PMC5215795

[11] Scapagnini G Marchegiani A Rossi G Zago M Jowarska J Wael M. Management of all three phases of wound healing through the induction of fluorescence biomodulation using fluorescence light energy. Photonic Diagnosis Treat Infect Inflamm Dis II. (2019) 31. 10.1117/12.2508066. [Epub ahead of print].

[12] Marchegiani A Fruganti A Spaterna A Cerquetella M Tambella AM Paterson S. The effectiveness of fluorescent light energy as adjunct therapy in canine deep pyoderma: a randomized clinical trial. Vet Med Int. (2021) 2021:6643416. 10.1155/2021/6643416 33505646 PMC7811420

[13] Marchegiani A Tambella AM Fruganti A Spaterna A Cerquetella M Paterson S. Management of canine perianal fistula with fluorescence light energy: preliminary findings. Vet Dermatol. (2020) 31:460–e122. 10.1111/vde.12890 32914496

[14] Apostolopoulos N Mayer U. Use of fluorescent light energy for the management of bacterial skin infection associated with canine calcinosis cutis lesions. Vet Rec Case Rep. (2020) 8:1285. 10.1136/vetreccr-2020-001285

[15] Marchegiani A Spaterna A Cerquetella M Tambella AM Fruganti A Paterson S. Fluorescence biomodulation in the management of canine interdigital pyoderma cases: a prospective, single-blinded, randomized and controlled clinical study. Vet Dermatol. (2019) 30. 10.1111/vde.12785 31407840

[16] Hill PB Lo A Eden CAN Huntley S Morey V Ramsey S. Survey of the prevalence, diagnosis and treatment of dermatological conditions in small animals in general practice. Vet Rec. (2006) 158:533–9. 10.1136/vr.158.16.533 16632525

[17] Beco L Guaguère E Lorente Méndez C Noli C Nuttall T Vroom M. Suggested guidelines for using systemic antimicrobials in bacterial skin infections (2): antimicrobial choice, treatment regimens and compliance. Vet Rec. (2013) 172:156–60. 10.1136/vr.101070 23292948 PMC3582090

[18] Duclos DD Hargis AM Hanley PW. Pathogenesis of canine interdigital palmar and plantar comedones and follicular cysts, and their response to laser surgery. Vet Dermatol. (2008) 19:134–41. 10.1111/j.1365-3164.2008.00662.x 18477329

[19] Miller W Griffin C Campbell K. Muller & Kirk's Small Animal Dermatology. 7th ed. St. Louis, MO: Elsevier (2013).

[20] Linek M Favrot C. Impact of canine atopic dermatitis on the health-related quality of life of affected dogs and quality of life of their owners. Vet Dermatol. (2010) 21:456–62. 10.1111/j.1365-3164.2010.00899.x 20492625

[21] Noli C Colombo S Cornegliani L Ghibaudo G Persico P Vercelli A. Quality of life of dogs with skin disease and of their owners. Part 2: administration of a questionnaire in various skin diseases and correlation to efficacy of therapy. Vet Dermatol. (2011) 22:344–51. 10.1111/j.1365-3164.2011.00956.x 21435044

[22] Noli C Minafò G Galzerano M. Quality of life of dogs with skin diseases and their owners. Part 1: development and validation of a questionnaire. Vet Dermatol. (2011) 22:335–43. 10.1111/j.1365-3164.2010.00954.x 21410569

[23] Nikolis A Fauverghe S Scapagnini G Sotiriadis D Kontochristopoulos G Petridis A. An extension of a multicenter, randomized, split-face clinical trial evaluating the efficacy and safety of chromophore gel-assisted blue light phototherapy for the treatment of acne. Int J Dermatol. (2018) 57:94–103. 10.1111/ijd.13814 29152718

[24] Crema A Scarpa C Sonda R Rizzato S Masciopinto G Bassetto F. Fluorescent light energy and chronic lesions: a winning association. Plast Reconstr Surg Glob Open. (2021) 9. 10.1097/GOX.0000000000003667. [Epub ahead of print]. 34277317 PMC8277272

